# Purification, Structural Characterization, and Bioactivity of *Amaranthus hypochondriacus* Lectin

**DOI:** 10.3390/molecules29215101

**Published:** 2024-10-29

**Authors:** Maria Fernanda Resendiz-Otero, Aurea Bernardino-Nicanor, Olivia Lugo-Magaña, Gabriel Betanzos-Cabrera, Leopoldo González-Cruz, José A. Morales-González, Gerardo Acosta-García, Eduardo Fernández-Martínez, Arturo Salazar-Campos, Carmen Valadez-Vega

**Affiliations:** 1 Departamento de Ingeniería Bioquímica, Instituto Tecnológico de Celaya, Av. Tecnológico y A. García Cubas S/N, Apartado Postal 57, Celaya 38010, Mexico; d2103025@itcelaya.edu.mx (M.F.R.-O.); aurea.bernardino@itcelaya.edu.mx (A.B.-N.); leopoldo.gonzalez@itcelaya.edu.mx (L.G.-C.); 2 Preparatoria Número 1, Universidad Autónoma del Estado de Hidalgo, Av. Benito Juárez S/N, Constitución, Pachuca de Soto 42060, Mexico; olivia_lugo@uaeh.edu.mx; 3 Área Académica de Nutrición, Instituto de Ciencias de la Salud, Universidad Autónoma del Estado de Hidalgo, Pachuca Hidalgo 42113, Mexico; gbetanzo@uaeh.edu.mx; 4 Laboratorio de Medicina de Conservación, Escuela Superior de Medicina, Instituto Politécnico Nacional, México, Plan de San Luis y Díaz Mirón, Col. Casco de Santo Tomás, Del. Miguel Hidalgo, Ciudad de Mexico 11340, Mexico; jmorales101@yahoo.com.mx; 5 Departamento de Ingeniería Bioquímica y Ambiental, Tecnológico Nacional de México/IT de Celaya, Antonio-García Cubas Pte #600 Esq. Av. Tecnológico, Celaya 38010, Mexico; gerardo.acosta@itcelaya.edu.mx; 6 Laboratory of Medicinal Chemistry and Pharmacology, Centro de Investigación en Biología de la Reproducción, Área Académica de Medicina, Instituto de Ciencias de la Salud, Universidad Autónoma del Estado de Hidalgo, Pachuca Hidalgo 42113, Mexico; 7 Área Académica de Medicina, Instituto de Ciencias de la Salud, Universidad Autónoma del Estado de Hidalgo, Pachuca Hidalgo 42113, Mexico

**Keywords:** *Amaranthus hypochondriacus*, lectin, purification

## Abstract

Lectin extracted from *Amaranthus hypochondriacus* was purified using an affinity column with an agarose-fetuin matrix specific to the lectin of interest. Purification was confirmed by SDS-PAGE, revealing a single protein band with a molecular mass of 34.4 kDa. A hemagglutination assay showed that the lectin had a higher affinity for human type A erythrocytes, and its hemagglutinating activity was inhibited only by fetuin, not by mono-, di-, or trisaccharides. This demonstrated the lectin’s selectivity for the N-acetylgalactosamine present on the surface of type A erythrocytes and fetuin. Amaranth lectin exhibited antioxidant activity, which was attributed to the phenolic compounds, amino acids, and specific peptides within the protein structure that are known for their antioxidant properties. Infrared (IR) spectroscopy provided a structural analysis and confirmed lectin glycosylation, a crucial factor in its stability and its ability to bind specific glycans on cell surfaces. Cu^2+^, Mn^2+^, and Zn^2+^ ions were found in the lectin, and these ions were strongly bound to the protein, as dialysis against ethylenediaminetetraacetic acid (EDTA) did not remove them. pH and temperature influenced lectin stability, with higher hemagglutinating activity observed at pH 7, and it remained thermostable at 25 °C.

## 1. Introduction

Lectins comprise a heterogeneous class of proteins and glycoproteins found in a wide variety of organisms, including plants, animals, fungi, and bacteria. They play significant roles in various biological processes within plants, such as defense [1]. These are non-immune origin proteins with the ability to recognize simple or complex carbohydrates, binding to them in a reversible and highly specific manner without covalently modifying the structures of the sugars to which they bind [2,3,4,5].

In the plant kingdom, these proteins are found in different parts of the plant, such as seeds, leaves, roots, and tubers. They are proteins that enter the secretory system, accumulating in vacuoles, the cell wall, and intercellular spaces. Phytohemagglutinins accumulated in the cotyledons are present at relatively high levels (1–8%) [1], contributing to the protection of the plant’s genetic material during growth [6]. Additionally, phytohemagglutinins are key in the defense of plants against pathogens. In the case of the common bean (*Phaseolus vulgaris*), lectins can bind to the surface of insects and microorganisms, inhibiting their growth or causing their death [7]. These proteins are involved in defense mechanisms and have demonstrated inhibitory effects on the growth of fungi and bacteria [8].

Lectins are classified according to species, sequence homology similarity, and carbohydrate-binding specificity or selectivity. Plant-derived lectins are grouped based on their carbohydrate recognition domain and classified into five groups: fucose, galactose/N-acetylgalactosamine, glucose/N-acetylglucosamine, mannose, and N-acetylneuraminic acid [5]. Based on sequence similarity, they are divided into eight families, including the amaranth lectin family [6].

The term “amaranthin” refers to the lectin purified from the seeds of *Amaranthus leucocarpus* [9] and *Amaranthus cruentus* [10]. These are homodimeric proteins composed of two subunits of approximately 33 kDa each. It has been reported that the soluble protein content of amaranth lectin ranges between 3% and 5%. This type of lectin exhibits attraction to N-acetylgalactosamine (GalNAc), although it shows significantly higher affinity for the disaccharide galactose beta 1,3 N-acetylgalactosamine (Galβ (1,3) GalNAc) [11].

The amino acid sequence of the lectin from *Amaranthus caudatus* has shown that the protomers consist of two domains of approximately 150 residues, with high similarity between them. Each domain has a tertiary fold characteristic of the B-trefoil protein family, suggesting a possible duplication and tandem insertion of a single ancestral domain. The carbohydrate-binding sites are located at the junction between the two domains, suggesting that the ancestral form of the protein may have been a single-domain structure. It has been demonstrated that each domain of the *Amaranthus caudatus* lectin consists of three subdomains with some residual sequence similarity, indicating that there are tandem duplications and insertions of a small polypeptide, approximately 50 residues in length and similar to the B-chain of type 2 RIP [12].

Amaranth lectins have been the subject of study in various biological research works, demonstrating their potential in regulating cellular processes. Studies have shown that these proteins are cytotoxic to certain cancer cell lines. Moreover, they can induce apoptosis in cancer cells and regulate cell adhesion [13,14]. The effects of these lectins on the modulation of gene expression have also been studied, revealing that they can regulate the expression of genes related to cell proliferation, apoptosis, and inflammation [15,16,17]. In addition to their biological significance, lectins have garnered interest in scientific research and biotechnology due to their potential applications in glycoprotein purification techniques and glycobiology studies and as tools for disease diagnosis and treatment [3].

Currently, the term “amaranthin-like” is used to group N-acetylgalactosamine-specific lectins that are closely related to this domain, such as the Hfr-2 lectin present in wheat (*Triticum aestivum*), which acts as a defense mechanism for the plant against Hessian fly larvae [18], or the lectin isolated from flaxseed (*Linum usitatissimum*), which exhibits the domain characteristic of the amaranthin-type lectin family, whose functions are related to defense [19].

Understanding the structure and function of lectins continues to advance, revealing more about their role in biology and their potential use in medicine and technology. In this study, we isolated and purified the lectin present in the seeds of *Amaranthus hypochondriacus*, evaluating its structure and biochemical properties, with the aim of contributing to the knowledge of plant proteins with potential uses in biomedicine and biotechnology. The purification and characterization of lectins are fundamental, as they ensure the accurate identification of their biological properties and guarantee their purity for therapeutic applications. Contributing to the understanding of their structure and binding mechanisms allows the development of targeted treatments and improves reproducibility in scientific studies, underscoring the importance of these processes in advancing knowledge and biotechnological innovation.

## 2. Results

**Figure 1:**
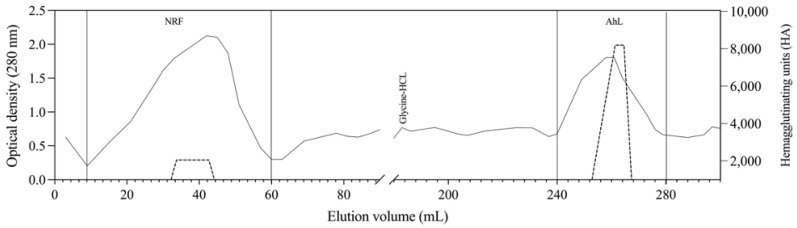
*Amaranthus hypochondriacus* lectin (AhL) purification on an agarose-fetuin affinity chromatography column. The non-retained fraction (NRF) was eluted with PBS (50 mM pH 7.4), and amaranth lectin (AhL) was eluted with glycine-HCl (50 mM, pH 2.5). Hemagglutination in blood group A erythrocytes (----).

The amaranth seed contained 69.6% total carbohydrates, 15.8% protein, 7.1% ash, 4.2% fat, and 3.3% moisture. Figure 1 shows the chromatogram of the lectin purification from *Amaranthus hypochondriacus* (AhL) by affinity chromatography following ammonium sulfate precipitation of an aqueous extract. The crude extract (CE) had a protein concentration of 150.5 mg/mL; after ammonium sulfate precipitation, the protein concentration in AhL was 17.2 mg/mL. The chromatogram (Figure 1) shows that lectin was recovered in a single peak after the addition of 50 mM glycine-HCl, pH 2.5, with a specific activity of 47,702 HA/protein, achieving a 17.5-fold purification, as shown in Table 1. An analysis of the carbohydrate content in AhL revealed a 4.6% content, suggesting that the lectin is a glycoprotein.

### 2.1. Identification of Purified Lectin from Amaranthus hypochondriacus Seeds by Electrophoresis (SDS-PAGE)

**Figure 2:**
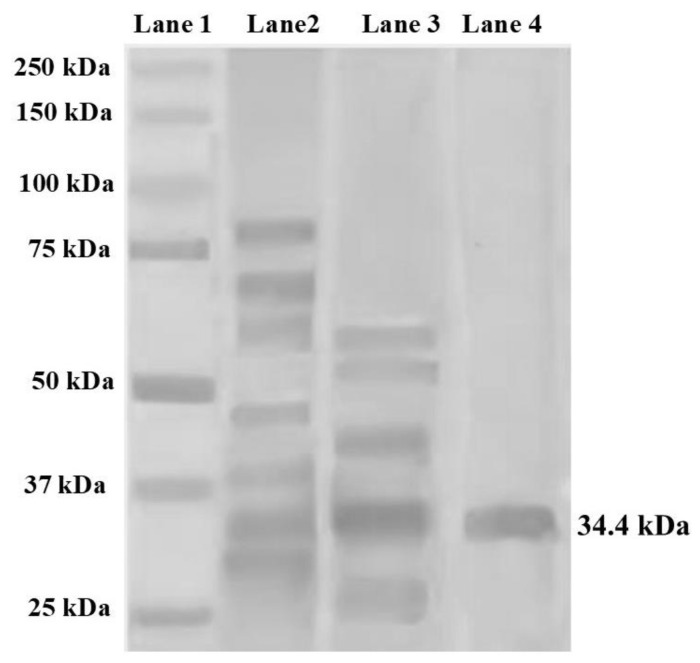
SDS-PAGE of *Amaranthus hypochondriacus* lectin. Lane 1, molecular weight marker; Lane 2, crude extract (CE); Lane 3, the non-retained fraction (NRF); and Lane 4, purified amaranth lectin obtained using an agarose-fetuin affinity chromatography column (AhL).

Figure 2 shows the SDS-PAGE electrophoresis of the purified lectin from *Amaranthus hypochondriacus* seeds. Lane 1 contains the molecular weight marker, while the subsequent lanes display the CE and fractions obtained after purification. In lane 4, a single band with a molecular weight of 34.4 kDa is observed, corresponding to the pure lectin from *Amaranthus hypochondriacus* seeds (AhL).

### 2.2. IR Spectroscopy

**Figure 3:**
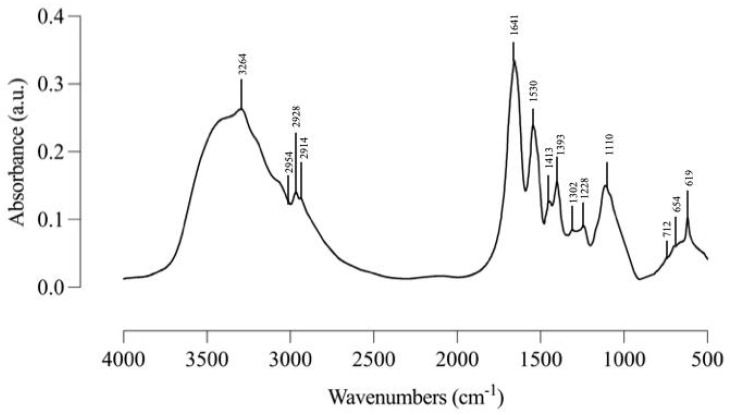
IR spectrum *Amaranthus hypochondriacus* lectin. The samples were pressed into KBr pellets with a sample/KBr ratio of 1:100.

The IR spectrum shown in Figure 3 was used to determine the type of functional groups and the secondary structure of a protein. The hydrogen bonds formed between the carbonyl group and amide are the main forces maintaining the protein’s secondary structure. The broad absorption band at 3264 cm^−1^ is a characteristic protein band, attributed to the overlap between OH vibrations. The three absorption bands between 3000 and 2900 cm^−1^ indicated asymmetric stretching vibration of methyl (–CH_3_), asymmetric stretching vibration of methylene (=CH_2_), and symmetric stretching vibration of methyl (–CH_3_), respectively. The absorption bands at 1641 cm^−1^ (amide I band) and 1530 cm^−1^ (amide II band) are important characteristic protein absorption bands, which indicated C=O stretching vibration, CN stretching vibration, and NH stretching vibration, respectively. The two absorption bands at 1500–1300 cm^−1^ were caused by C–H bending vibrations. Additionally, the N-glycosidic bond of the *Amaranthus hypochondriacus* seed lectin was revealed by the absorption band at 1110 cm^−1^. The three absorption bands between 800 and 600 cm^−1^ indicated the presence of metal ions within the protein structure.

### 2.3. Hemagglutination Assay

The specific hemagglutinating activity was evaluated across blood groups A, B, and AB. Table 2 shows that AhL exhibited higher hemagglutinating activity compared to the crude extract (CE), with both samples displaying a greater affinity for erythrocytes from blood group A.

### 2.4. Inhibition of Hemagglutinating Activity

It was found that only galactose and fetuin were able to inhibit AhL hemagglutination, whereas the monosaccharides, disaccharides, and other glycoproteins tested did not affect lectin hemagglutinating activity (Table 3).

### 2.5. Total Phenolic Content and Antioxidant Capacity

The concentration of phenolic constituents was determined, showing the highest concentration in the crude extract (83.7 ± 0.95 mg EAG/mg), with a significant decrease (*p* < 0.05) during the AhL purification process, reducing by 84% from the crude extract to AhL (13.86 ± 1.16 mg EAG/mg).

Table 4 and Table 5 illustrate the radical scavenging capacity against ABTS•^+^ and DPPH•, with the IC_50_ (IC_50_ mean inhibitory concentration) values significantly increasing during the purification steps (*p* < 0.05), rising from 102.5 to 156.8 mg/mL for ABTS•^+^ and from 177.4 to 322.3 mg/mL for DPPH•. Furthermore, the scavenging percentages in both studies increase with the rising concentrations in each test, indicating a direct relationship between concentration and radical scavenging capacity.

A Pearson correlation coefficient analysis between the antioxidant activity assays and phenolic constituents showed a statistically significant correlation (*p* < 0.05) between the antioxidant activity assay (ABTS•^+^) and the phenols (R^2^ = 0.974) present in the retained fraction.

### 2.6. pH and Temperature Stability

**Figure 4:**
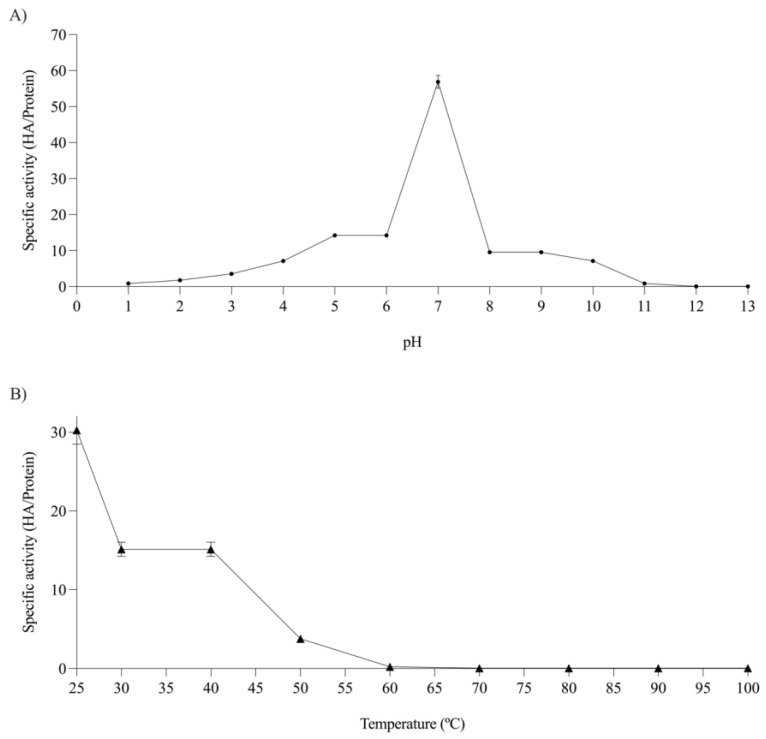
(**A**) pH stability, and (**B**) thermal stability of AhL.

Hemagglutination activity was determined across a pH range of 1 to 13. The highest activity was observed at pH 7. Both acidic and basic conditions resulted in decreased activity, with no activity detected at pH values below 2 or above 11 (Figure 4A). On the other hand, the effect of temperature on hemagglutinating activity was determined, and it was found that the optimal temperature was 25 °C. The gradual increase in temperature led to a decrease in activity until a complete loss of activity was observed at 60 °C (Figure 4B).

### 2.7. Metal Ion Content and Effect of Ion Metal on Hemagglutination

The results of plasma spectrometry, shown in Table 6, indicate that in the native lectin, Mn^2+^, Cu^2+^, Ca^2+^, and Zn^2+^ are present in higher concentrations compared to K^1+^ and Na^+1^. After dialyzing AhL vs. H_2_O, it was observed that Na^1+^, K^1+^, Cu^2+^, and Zn^2+^ did not vary with respect to the native AhL, but the contents of Mg^2+^, Mn^2+^, and Ca^2+^ decreased and were higher than that of calcium.

In relation to the lectin dialyzed against EDTA for 24 h, Na¹⁺ showed no change; however, all the other ions decreased significantly compared to the native lectin. In general, the reduction in ions was especially notable in the samples dialyzed in EDTA, a chelating agent that facilitated a more efficient removal of metals compared to dialysis against H_2_O for 24 h; the exception was the Ca^2+^, Mg^2+^ and Mn^2+^ ions, which were more attracted to water than to EDTA.

After 24 h of dialysis, a decrease in the hemagglutinating activity of the lectin was observed. Dialysis against water resulted in a specific activity of 238.5 HU/protein, while dialysis with EDTA produced an activity of 29.8 HU/protein. In contrast, the control group exhibited 477.1 HU/protein.

### 2.8. Hemolytic Activity of AhL

**Figure 5:**
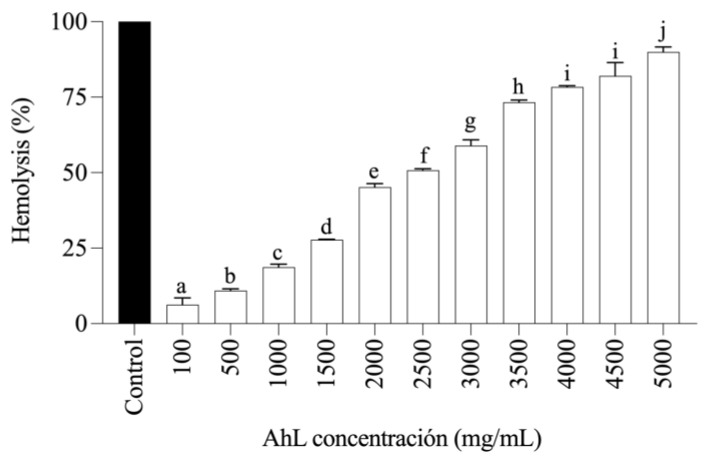
Hemolytic activity of AhL on human erythrocytes A compared to the control (Triton X-100). AhL: *Amaranthus hypochondriacus* lectin. Different letters (a–j) indicate significant differences between concentrations (*p* < 0.05). The values represent mean ± standard deviation (SD) of three independent experiments.

Figure 5 shows the hemolytic activity of AhL compared to the Triton X-100 control. It was observed that the highest concentration tested, 5000 mg/mL, did not achieve 100% hemolysis. The half-hemolytic concentration (HC_50_) was determined to be 2461.3 mg/mL. These results suggest that, although the lectin exhibits hemolytic activity, its effectiveness is lower than that of the control.

## 3. Discussion

This study performed a partial purification and characterization of the lectin from *Amaranthus hypochondriacus*, a seed that is widely distributed and commonly included in the diet of various cultures [20]. A proximal chemical analysis was conducted, and the results were consistent with those reported for the Amaranthus species, highlighting that the protein content is higher than that of other cereals, such as maize, rye, and rice [21]. The observed variations can be attributed to the origin of the seed, the variety, and the differences in climatic conditions during cultivation, as well as in storage and processing [22,23].

The lectin extracted from this species represents an optimal raw material for the isolation of phytohemagglutinins. For purification, an affinity column with an agarose-fetuin matrix, specific to the lectin of interest, was employed [24]. This type of column has previously been used for the purification of lectins from *Amaranthus leucocarpus* [25] and *Phaseolus coccineus* [26]. The chromatographic patterns observed in this study differ considerably from those obtained for other Amaranthus species; this is likely due to the fact that the recovery values are closely related to the type of column used and its affinity, as well as the methodology applied and the plant material employed [11,25,27].

To confirm the purity of the lectin, an analysis using SDS-PAGE gel electrophoresis was conducted, where a single band was observed in the retained fraction, with a molecular mass of 34.4 kDa. The alignment of this molecular mass with previous studies suggests that the lectin is of the amaranth type [4,5,11,28,29].

Additionally, infrared spectroscopy was performed, providing a structural analysis of the lectin [30] and testing lectin glycosylation. The glycosylation of lectins not only affects their stability and solubility but also influences their ability to recognize and bind to specific glycans on cell surfaces. This process is essential for proper cell signaling, immune response, and cell adhesion [4]. In this study, the band at 1110 cm^−1^ in the IR spectrum was found to correspond to the glycosylation of amaranth lectin, which is consistent with reports from other authors indicating that *Amaranthus hypochondiacus* lectin is glycosylated [27,31].

The ability of the lectins to induce hemagglutination was evaluated to determine their specificity towards certain types of erythrocytes, showing selectivity for human type A erythrocytes due to the presence of N-acetylgalactosamine (GalNAc) on their cell surfaces [32]. The results obtained are within the range of values previously reported in the literature [9,25,27,33]. Evaluating hemagglutination activity is crucial for understanding how lectins interact with different cell types, allowing for the identification of specific glycan patterns on their surfaces, which highlight particularly significant traits within the amaranth lectin family [6,32].

Antioxidant activity is essential in both biomedicine and biotechnology due to its ability to protect against cellular damage caused by reactive oxygen species (ROS). The oxidative balance of the body is influenced by diet [34,35]. Among the nutrients with the highest antioxidant potential are vitamins, while non-nutrient compounds of phenolic origin and antioxidant peptides also stand out [36,37,38]. Our findings align with other studies that reported antioxidant activity in amaranth, which was primarily due to the presence of phenolic compounds or peptides [39]. The observed antioxidant function in AhL may be related to the amino acids present in its structure. Certain plant proteins can inhibit lipid oxidation through various pathways, neutralizing reactive oxygen species and other free radicals, chelating transition metals, and reducing hydroperoxides [40,41,42,43].

Amino acids such as Tyr, Trp, Met, Lys, Cys, and His exhibit antioxidant activity, and peptides containing active fragments like Tyr-His-Tyr and Pro-His-His have been reported to effectively stabilize ROS [40,44,45,46]. Amaranth has been reported to contain high concentrations of essential amino acids, such as Lys, Phe/Tyr, Met/Cys, and Trp, with three potential antioxidant peptide sequences identified through in silico analysis [47,48].

The effect of pH on hemagglutinating activity was analyzed, revealing that lectins retain their activity only within pH ranges close to neutrality [9,28,31,49]. These changes in lectin activity suggest that the side chain environment of aromatic residues and/or their orientation is significantly altered when the pH of the medium changes. This alters the protein’s ability to bind to carbohydrates, which can lead to a decrease in or loss of hemagglutinating activity, potentially resulting in lectin denaturation and impaired function [50,51].

The effect of temperature on hemagglutinating activity was also examined. In the range of 20 to 70 °C, the lectins did not lose their biological function, which is consistent with the results from other studies [9,10,49,52]. These observations suggest that the secondary structure of lectins undergoes variations as the temperature increases, leading to denaturation and ultimately affecting the protein’s biological activity [50]. The thermal resistance of lectins is a controversial topic. From a nutritional perspective, lectin resistance to high temperatures is undesirable, as lectins are considered antinutritional factors, causing adverse effects such as intestinal epithelial alterations and interference with nutrient absorption. However, from a biotechnological standpoint, thermal stability is crucial as it ensures that bioactive substances maintain their efficacy throughout all phases, from production to final use [53]. Oligosaccharides protect glycoproteins from physicochemical damage, reinforcing their structure through electrostatic interactions and non-covalent bonds, while some disulfide bridges contribute to thermal stability [54].

AhL is a glycoprotein that contains 4.6% carbohydrate content; this is consistent with reports on lectin from *Amaranthus viridis* [55]. In contrast, lectins from *Amaranthus caudatus* have been reported to lack sugar residues [11].

Some lectins are characterized by the presence of metal ions, which are crucial for their biochemical activity and functional properties. The loss of hemagglutinating activity after 24 h of dialysis against water may indeed be due to the depletion of these metal ions.

As we confirmed, AhL is a metalloprotein that requires the presence of metal ions to properly induce hemagglutination. As the ion concentration decreased during dialysis, the hemagglutinating activity was significantly reduced.

It is known that demetallization induces structural changes in lectins in the metal-binding regions, compromising their interactions with carbohydrates and reducing their biological activity, without affecting the protein’s secondary structure [56].

It has been reported that in some lectins, such as those from *Amaranthus caudatus* and *Amaranthus leucocarpus*, metal ions are not necessary for their hemagglutinating action. Although this activity may be diminished, their biological activity remains intact [9,11,33]. However, the lectin from *Amaranthus hypochondriacus* that we studied showed that the reduction in metal ions significantly affected its biological activity. Proteins, such as lectins, can act as chelating agents by binding metal ions through functional groups, like carboxyl, amino, or thiol groups, which form stable complexes with the metals. This mechanism is crucial in modulating the reactivity of metal ions or facilitating their removal from biological systems. For instance, the role of proteins as chelators in the regulation of essential metals and the detoxification of harmful metal ions has been thoroughly examined. Calcium was extracted more effectively with water than with EDTA. Water may dissolve or release loosely bound calcium ions from the lectin without forming strong chelation complexes. In contrast, EDTA, being a stronger chelating agent, may preferentially bind other metal ions in the solution or within the lectin, resulting in less calcium removal [56].

The lectin induced hemolytic activity in type A human erythrocytes. However, the values were concentration-dependent, with low activity classified as between 0 and 40%, moderate activity as between 40 and 80%, and high activity at ≥80%. These data corroborate the findings of other authors, demonstrating that lectins at low concentrations cause minimal hemolysis in human erythrocytes. It is crucial to study the hemolytic effects on erythrocytes, as hemolysis is an undesirable outcome when working with lectins that exhibit biological activity [52].

## 4. Materials and Methods

### 4.1. Plant Material

The seeds of *Amaranthus hypochondriacus* were obtained from a local producer in the Xochimilco borough of Mexico City (19°25′50.5″ N–99°01′13.4″ W). A selection process was carried out to remove foreign matter and damaged grains. The seeds were ground using an IKA A10 grain mill (IKA-Werke GmbH & Co. KG, Wilmington, NC, USA). The resulting flour was stored in a commercial refrigerator at 4 °C until use.

### 4.2. Human Cells

Human erythrocytes A, B, and AB were obtained from healthy voluntary donors who provided informed consent, in accordance with the Mexican Official Standards NOM-007-SSA3-2011 [57] and NOM-253-SSA1-2012 [58]. The study was conducted in accordance with the Declaration of Helsinki. All procedures received approval from the Ethics and Research Committee of the Autonomous University of the State of Hidalgo under the reference number CEEI-000011-2019, on 9 January 2019.

### 4.3. Proximate Chemical Analysis

The proximate analysis was performed using the official methods of the Association of Analytical Communities (AOAC) [59]. The methods used included 950.46 for moisture, 960.34 for fat, and 920.153 for ash, and the total protein was determined by the Kjeldahl method (928.08) using a nitrogen conversion factor of 5.85 [60]. The total carbohydrate content was calculated by difference.

### 4.4. Extraction and Purification of Lectin

Lectin from *Amaranthus hypochondriacus* (AhL) was extracted following the method described by Valadez-Vega et al. [61]. The flour was suspended in phosphate-buffered saline (PBS, 10 mM) at a ratio of 1:10 (*w*/*v*) for 16 h at 4 °C. The mixture was then centrifuged at 10,000 rpm for 60 min at 4 °C. Proteins were precipitated from the supernatant with 80% (NH_4_)_2_SO_4_ at 4 °C, followed by centrifugation at 10,000 rpm for 60 min at 4 °C. The precipitate was suspended in PBS and dialyzed against PBS with four changes over 24 h. The obtained proteins, or crude extract (CE), were subjected to lectin purification by affinity chromatography using a fetuin-agarose matrix column (Sigma Chemical Co., Burlington, MA, USA).

Prior to sample injection, the affinity column (3 × 20 cm, Sigma Aldrich, St. Louis, MO, USA) was equilibrated with 20 volumes of PBS. Three milliliters of the sample were applied, and the non-retained fraction was washed with 20 volumes of PBS. The bound fraction, corresponding to the lectin and showing hemagglutination (AhL), was eluted using glycine-HCl (50 mM, pH 2.5). The collected fractions were dialyzed against distilled water at 4 °C and lyophilized for storage at 4 °C.

### 4.5. Protein Quantification

Protein concentration was determined using the Bradford method [62], with bovine serum albumin (Sigma Chemical Co., Burlington, MA, USA) as the standard.

### 4.6. Sodium Dodecyl Sulphate Polyacrylamide Gel Electrophoresis (SDS-PAGE)

The crude extract (CE) and pure lectin were evaluated by polyacrylamide gel electrophoresis (SDS-PAGE) in a vertical Mini-Protean II electrophoresis system at 110 mA (Bio-Rad, Hercules, CA, USA) with 12% gel, according to Laemmli. The samples were solubilized in sample buffer (80 mM Tris-HCl, pH 6.8, 10% glycerol, 0.02% bromophenol blue, and 2% SDS) to a final concentration of 4 mg/mL. The proteins were stained with a 0.12% Coomassie Brilliant Blue R-250 solution, and excess dye was removed by washing the gel with hot distilled water. Separated proteins were visualized by Coomassie Brilliant Blue R-250 staining (Sigma Chemical Co., Burlington, MA, USA). The molecular weight marker used was Precision Plus Protein Standards All Blue, catalog number #161-0373 (Bio-Rad, Hercules, CA, USA). To calculate the molecular weight, a standard calibration curve was generated by measuring the migration distances of the molecular weight marker bands in centimeters. The molecular weight of the purified lectin was calculated based on its migration distance.

### 4.7. Infrared Spectroscopy (IR)

Infrared spectroscopy was performed on the fractions obtained during the purification of AhL. The analysis was conducted using a Perkin Elmer IR spectrophotometer (Perkin Elmer, Inc., Waltham, MA, USA). The samples were prepared as KBr pellets, using a mixture of the lyophilized sample and potassium bromide (KBr) discs (Sigma Chemical Co., Burlington, MA, USA). One hundred milligrams of KBr was mixed with 1 mg of the sample, which was previously finely ground in an agate mortar. The KBr and sample mixture was transferred to a vacuum chamber die, allowing the removal of air or fluids between the grains, resulting in a compressed pellet at a pressure of 500–1000 kg/cm^2^. Spectra were recorded in absorbance mode in the range of 4000 to 400 cm^−1^ [63].

### 4.8. Lectin Hemagglutination

The hemagglutination assay was conducted according to the methodology proposed by Valadez-Vega et al. [64]. A 96-well U-bottom microplate was used. Each well was filled with 50 µL of PBS; 50 µL of the sample (1 mg/mL) was added to the first well, followed by two-fold serial dilutions. Then, 50 µL of 2% erythrocyte suspension was added to each well. The mixture was allowed to react for 60 min at room temperature, and the last well showing hemagglutination was recorded. The results were reported as specific activity (HU/protein).

### 4.9. Hemagglutination Inhibition

For the determination of hemagglutination inhibition, 96-well U-shaped microplates were used. Each well was filled with 50 µL of PBS. To the first well, 50 µL of sugar solution (50 mM) was added: galactose, fetuin, dextrose, ovalbumin, fructose, glucose, levulose, mannose, ribose, xylose, maltose, arabinose, raffinose, trehalose, sucrose, lactose (Sigma Chemical Co., Burlington, MA, USA). Subsequently, 50 µL of AhL solution (0.1 mg/mL) was added to each well and incubated for 1 h at room temperature, followed by the addition of 50 µL of 2% human erythrocyte suspension. It was incubated for 1 h at room temperature, and the minimum sugar dilution that caused hemagglutination was observed. The results were reported as the maximum sugar dilution causing hemagglutination [64].

### 4.10. Phenolic Compound Content

The phenolic compound content was determined following the method described by Singleton et al. [65]. To 100 µL of AhL solution (1 mg), 1 mL of water was added, followed by 500 µL of Folin–Ciocalteu reagent (1:10) (Sigma Chemical Co., Burlington, MA, USA) and 400 µL of Na_2_CO_3_ (7.5%, Sigma Chemical Co., Burlington, MA, USA). The reaction was left in the dark for 30 min, and absorbance was measured at 765 nm (Epoch 2 spectrophotometer, BioTek, Winooski, VT, USA). A standard curve was prepared using gallic acid (Sigma Chemical Co., Burlington, MA, USA). The results were reported as mg gallic acid equivalents per mg of sample (mg GAE/mg).

### 4.11. Antioxidant Capacity

#### 4.11.1. ABTS•^+^ Assay

The assay was conducted following the methodology described by Re et al. An aliquot of 100 µL of AhL (1 mg/mL in water) was added to 900 µL of ABTS•+ solution (Sigma Chemical Co., Burlington, MA, USA), as previously prepared. The reaction was al-lowed to proceed for 5 min in the dark, and absorbance was measured at 734 nm using an Epoch 2 spectrophotometer (BioTek, Winooski, VT, USA). A standard curve with Trolox (Sigma Chemical Co., Burlington, MA, USA) was used; the results were reported as % scavenging, and the IC_50_ (mg/mL) was calculated.

#### 4.11.2. DPPH• Assay

To 100 µL of AhL solution (1 mg/mL), 900 µL of DPPH• solution (Sigma Chemical Co., Burlington, MA, USA) was added, and the mixture was allowed to react in the dark for 1 h. Absorbance was measured at 520 nm using an Epoch 2 spectrophotometer (BioTek, Winooski, VT, USA). A standard curve of Trolox (Sigma Chemical Co., Burling-ton, MA, USA) was prepared, and the results were expressed as % scavenging, with the IC_50_ (mg/mL) calculated accordingly.

### 4.12. Effect of pH and Temperature on Hemagglutination

#### 4.12.1. Effect of pH

The pH was varied from 3 to 13 using the following 2 mM buffer solutions: sodium acetate (pH 3, 4, and 5), sodium phosphate (pH 6 and 7), Tris-HCl (pH 8), and glycine-NaOH (pH 9, 10, 11, 12, and 13). The samples were incubated for 60 min, followed by dialysis against PBS for 12 h at 4 °C. The hemagglutination test was performed using human erythrocytes type A. The AhL solution was prepared in PBS (1 mg/mL) [26].

#### 4.12.2. Effect of Temperature

Aliquots of 3 mL of AhL diluted in H_2_O were heated in a water bath at 25, 30, 40, 50, 60, 70, 80, 90, and 100 °C for 30 min, then immediately cooled. The hemagglutination test was performed using human erythrocytes type A [26].

### 4.13. Total Carbohydrate Content

The carbohydrate content in the AhL was determined using the phenol-sulfuric acid method described by Dubois et al. [66], with glucose (Sigma Chemical Co., Burlington, MA, USA) as the standard.

### 4.14. Metal Ion Content

The concentration of metals in the native and demetallized lectin was determined. For demetallization, two lectin samples were used, one sample was dialyzed against deionized water and the other against ethylenediaminetetraacetic acid (EDTA 0.02M) in NaCl (15 mM) pH 8, for 24 h. The native and demetallized lectin samples were digested with HNO_3_ (38%).

The metal ion content was determined by ICP/OES 8300 plasma spectrophotometry (Perkin-Elmer Optima™ 8300, Waltham, MA, USA), using a standard calibration curve for each ion (Ca^2+^, Mg^2+^, Mn^2+^, Cu^2+^, Na^1+^, K^1+^, and Zn^2+^). The concentration of the ions in the samples was then calculated by graphical interpolation [64].

### 4.15. Effect of AhL Demetallization on Hemagglutination

After the lectin was dialyzed against water or EDTA pH 8 (2 mM) in NaCl (15 mM), the hemagglutination test was performed using human erythrocytes type A, as mentioned above. The results were reported as specific activity (HU/protein).

### 4.16. Hemolytic Activity

Aliquots of 20 µL of type A erythrocyte suspension (4% in PBS) were mixed with AhL solution at different concentrations (100, 500, 1000, 1500, 2000, 2500, 3000, 3500, 4000, 4500, and 5000 µg/mL in PBS). The samples were incubated for 30 min at 37 °C, and absorbance was measured at 540 nm using an Epoch 2 spectrophotometer (BioTek, Winooski, VT, USA) [67]. Triton-X 100 (Sigma Chemical Co., Burlington, MA, USA) at 20% was used as a control. The results were expressed as a percentage of hemolysis, with the control considered as 100% hemolysis.

### 4.17. Statistical Analysis

All analyses were performed in triplicate, and the results are presented as mean ± standard deviation. The data were stored and statistically analyzed using ANOVA and Tukey’s test (*p* < 0.05) with GraphPad Prism 10 software (GraphPad Software, San Diego, CA, USA).

## 5. Conclusions

This research provides a comprehensive characterization of the lectin extracted from *Amaranthus hypochondriacus*, revealing its substantial biochemical and functional properties, which underscore its potential applications in nutrition and biotechnology. The partial purification of the lectin demonstrated a high protein content compared to traditional cereals, emphasizing the nutritional value of amaranth seeds. By utilizing an affinity column with an agarose-fetuin matrix, a purified lectin with a molecular mass of 34.4 kDa was successfully obtained and was consistent with previous reports on amaranth lectins. Infrared spectroscopy confirmed its glycosylation, a crucial factor for its binding interactions with specific glycans on cell surfaces. The lectin exhibited selectivity for human type A erythrocytes and demonstrated antioxidant activity, which was linked to specific amino acids and phenolic compounds.

The study also examined the effects of pH and temperature on hemagglutination activity, showing that the lectin maintains functionality across a range of conditions, although care must be taken to prevent denaturation. The presence of metal ions plays a crucial role in the biological activity of lectins, as evidenced by the significant impact observed in *Amaranthus hypochondriacus*. Unlike lectins from other amaranth species, AhL contains carbohydrates in its structure, and metal ions have proven to be crucial for its biological function. This highlights the importance of metal ions in maintaining the structural integrity and biological efficacy of lectins.

Understanding the biochemical characteristics of *Amaranthus hypochondriacus* lectin is critical, as they may provide insights into the potential applications of this protein in research areas such as cancer, immunology, and diabetes and as a diagnostic tool for various diseases. The lectin from Amaranthus hypochondriacus demonstrates potential as a biomolecule with health benefits and industrial applications, highlighting the necessity for further investigation into its mechanisms of action and possible uses across diverse fields.

## Figures and Tables

**Table 1:** Purification of *Amaranthus hypochondriacus* lectin (AhL).

Fraction	Protein (mg/mL)	Hemagglutinating Units (HA)	Specific Activity (HA/Protein) *	Purification Factor
CE	150.5	4096	27.2	1
AhL	17.2	8192	477.2	17.5

CE, crude extract; AhL, *Amaranthus hypochondriacus* lectin purified. *: Against human A erythrocytes.

**Table 2:** Hemagglutination activity of pure lectin human erythrocytes.

	Erythrocytes (HA/Protein)		
Sample	A	B	AB
CE	27.2 ± 1.1 ^a^	6.8 ± 0.5 ^b^	13.6 ± 0.9 ^c^
AhL	487.5 ± 0.7 ^a^	60.8 ± 1.3 ^b^	243.1 ± 0.6 ^c^

CE: crude extract; AhL: *Amaranthus hypochondriacus* seed lectin. Means with different superscripts are significantly different (Tukey’s test, *p* < 0.05).

**Table 3:** Effect of carbohydrates on the hemagglutination activity of AhL.

Monosaccharide/ Glycoprotein	Inhibitory Concentration * (µg/mL) AhL
Galactose	1.6
Fetuin	0.8

* lowest concentration to reach complete inhibition. AhL, *Amaranthus hypochondriacus* lectin.

**Table 4:** ABTS•^+^ radical scavenging capacity and IC_50_.

Concentration (mg/mL)	CE	AhL	Trolox
	Scavenging (%)		
0	43.63	45.46	16.07
125	52.18	45.28	16.73
250	60.97	55.57	19.88
500	65.82	68.08	30.74
1000	69.67	76.02	35.96
2000	82.18	96.00	39.21
IC_50_	102.54	156.76	7.12

AhL, *Amaranthus hypochondriacus* lectin; CE, crude extract; IC_50_, mean inhibitory concentration.

**Table 5:** DPPH• radical scavenging capacity and IC_50_.

Concentration (mg/mL)	CE	AhL	Trolox
	Scavenging (%)		
0	30.67	30.61	27.08
125	44.12	17.35	131.45
250	55.84	45.28	196.19
500	77.07	62.48	252.89
1000	96.27	87.87	288.43
2000	109.62	107.90	378.56
IC_50_	177.45	322.3	47.3

AhL, *Amaranthus hypochondriacus* lectin; CE, crude extract; IC_50_, mean inhibitory concentration.

**Table 6:** Metal content in AhL.

Lectin	Concentration (ppm)						
	Mg^2+^	K^1+^	Na^1+^	Cu^2+^	Mn^2+^	Ca^2+^	Zn^2+^
Native	138.3 ± 2.1	96 ± 2.2	55.1 ± 4.2	186.2 ± 0.3	196.2 ± 0.3	138.3 ± 2.1	185.3 ± 0.1
Dialyzed vs. H_2_O	119.5 ± 1.0 *	94.1 ± 0.4	46 ± 2.2	174.5 ± 4.1	186.2 ± 0.3 *	70.4 ± 2.1 *	182.6 ± 1.5
Dialyzed vs. EDTA	127.3 ± 2.1 *	77.8 ± 2.1 *	53.7 ± 1.8	166 ± 4.0 *	156.5 ± 1.7 *	127.1 ± 3.2 *	157.1 ± 1.7 *

The values represent mean ± standard deviation (SD) of three independent experiments. * Indicated significant differences against the native samples (*p* < 0.05). AhL: *Amaranthus hypochondriacus* lectin.

## Data Availability

Data is contained within the article.

## References

[1] Chrispeels M.J., Raikhel N.V. (1991). Lectins, Lectin Genes, and Their Role in Plant Defense. Plant Cell.

[2] Bah C.S.F., Fang E.F., Ng T.B. (2013). Medicinal Applications of Plant Lectins. Antitumor Potential and other Emerging Medicinal Properties of Natural Compounds.

[3] Kennedy J.F., Palva P.M.G., Corella M.T.S., Cavalcanti M.S.M., Coelho L.C.B.B. (1995). Lectins, Versatile Proteins of Recognition: A Review. Carbohydr. Polym..

[4] Sharon N. (2004). History of Lectins: From Hemagglutinins to Biological Recognition Molecules. Glycobiology.

[5] Van Damme E.J.M., Lannoo N., Peumans W.J. (2008). Plant Lectins.

[6] Van Damme E.J.M., Peumans W.J., Barre A., Rougé P. (1998). Plant Lectins: A Composite of Several Distinct Families of Structurally and Evolutionary Related Proteins with Diverse Biological Roles. CRC Crit. Rev. Plant Sci..

[7] Gatehouse A.M.R., Dewey F.M., Dove J., Fenton K.A., Pusztai A. (1984). Effect of Seed Lectins from Phaseolus Vulgaris on the Development of Larvae of Callosobruchus Maculatus; Mechanism of Toxicity. J. Sci. Food Agric..

[8] Gautam A.K., Gupta N., Narvekar D.T., Bhadkariya R., Bhagyawant S.S. (2018). Characterization of Chickpea (*Cicer arietinum* L.) Lectin for Biological Activity. Physiol. Mol. Biol. Plants.

[9] Zenteno E., Ochoa J.-L. (1988). Purification of a Lectin from Amaranthus Leucocarpus by Affinity Chromatography. Phytochemistry.

[10] Koeppe S.J., Rupnow J.H. (1988). Purification and Characterization of a Lectin from the Seeds of Amaranth (*Amaranthus cruentus*). J. Food Sci..

[11] Rinderle S.J., Goldstein I.J., Matta K.L., Ratcliffe R.M. (1989). Isolation and Characterization of Amaranthin, a Lectin Present in the Seeds of *Amaranthus caudatus*, That Recognizes the T- (or Cryptic T)-Antigen. J. Biol. Chem..

[12] Transue T.R., Smith A.K., Mo H., Goldstein I.J., Saper M.A. (1997). Structure of Benzyl T-Antigen Disaccharide Bound to *Amaranthus caudatus* Agglutinin. Nat. Struct. Biol..

[13] Mengoni A., Quiroga A.V., Añón M.C. (2016). Purificación y Caracterización de Una Lectina de *Amaranthus hypochondriacus*, Un Compuesto Antiproliferativo. INNOTEC.

[14] Quiroga A.V., Barrio D.A., Añón M.C. (2015). Amaranth Lectin Presents Potential Antitumor Properties. LWT-Food Sci. Technol..

[15] Gómez-Henao W., Saavedra R., Chávez-Sánchez F.R., Lascurain R., Zenteno E., Tenorio E.P. (2021). Expression Dynamics of the O-Glycosylated Proteins Recognized by Amaranthus Leucocarpus Lectin in T Lymphocytes and Its Relationship With Moesin as an Alternative Mechanism of Cell Activation. Front. Immunol..

[16] Gorocica P., Lascurain R., Hemandez P., Porras F., Bouquelet S., Vazquez L., Zenteno E. (1998). Isolation of the Receptor for Amaranthus Leucocarpus Lectin from Murine Peritoneal Macrophages. Glycoconj. J..

[17] Urrea F., Zenteno E., Avila-Moreno F., Javier Sanchez-Garcia F., Zuñiga J., Lascurain R., Ortiz-Quintero B. (2011). Amaranthus Leucocarpus Lectin (ALL) Enhances Anti-CD3-Dependent Activation of Murine T Cells and Promotes Cell Survival. Immunol. Investig..

[18] Puthoff D., Sardesai N., Subramanya S., Nemacheck J., Williams C. (2005). Hfr-2, a Wheat Cytolytic Toxin-like Gene, Is Up-regulated by Virulent Hessian Fly Larval Feeding. Mol. Plant Pathol..

[19] Faruque K., Begam R., Deyholos M.K. (2015). The Amaranthin-Like Lectin (LuALL) Genes of Flax: A Unique Gene Family with Members Inducible by Defence Hormones. Plant Mol. Biol. Rep..

[20] Rastogi A., Shukla S. (2013). Amaranth: A New Millennium Crop of Nutraceutical Values. Crit. Rev. Food Sci. Nutr..

[21] Venskutonis P.R., Kraujalis P. (2013). Nutritional Components of Amaranth Seeds and Vegetables: A Review on Composition, Properties, and Uses. Compr. Rev. Food Sci. Food Saf..

[22] Berganza B.E., Moran A.W., Guillermo Rodríguez M., Coto N.M., Santamaría M., Bressani R. (2003). Effect of Variety and Location on the Total Fat, Fatty Acids and Squalene Content of Amaranth. Plant Foods Human. Nutr..

[23] Schofield E.J., Rowntree J.K., Paterson E., Brewer M.J., Price E.A.C., Brearley F.Q., Brooker R.W. (2019). Cultivar Differences and Impact of Plant-Plant Competition on Temporal Patterns of Nitrogen and Biomass Accumulation. Front. Plant Sci..

[24] Kornfeld R., Kornfeld S. (1985). Assembly of Asparagine-Linked Oligosaccharides. Annu. Rev. Biochem..

[25] Hernández P., Bacilio M., Porras F., Juarez S., Debray H., Zenteno E., Ortiz B. (1999). A Comparative Study on the Purification of the *Amaranthus leucocarpus* Syn. *hypocondriacus* Lectin. Prep. Biochem. Biotechnol..

[26] González-Cruz L., Valadez-Vega C., Juárez-Goiz J.M.S., Flores-Martínez N.L., Montañez-Soto J.L., Bernardino-Nicanor A. (2022). Partial Purification and Characterization of the Lectins of Two Varieties of *Phaseolus coccineus* (Ayocote Bean). Agronomy.

[27] Ozeki M., Kamemura K., Moriyama K., Itoh Y., Furuichi Y., Umekawa H., Takahashi T. (1996). Purification and Characterization of a Lectin from *Amaranthus hypochondriacus*. Mexico Seeds. Biosci. Biotechnol. Biochem..

[28] Calderon de la Barca A., Vazquez-Moreno L. (1988). *Amaranthus cruentus* Lectin: Purification, Stability, and Some Biochemical Properties. J. Food Biochem..

[29] Liener I.E. (1991). From Soybeans to Lectins: A Trail of Research Revisited. Carbohydr. Res..

[30] Barth A. (2007). Infrared Spectroscopy of Proteins. Biochim. Biophys. Acta (BBA)-Bioenerg..

[31] Hasan I., Rahman S.N., Islam M.M., Ghosh S.K., Mamun M.R., Uddin M.B., Shaha R.K., Kabir S.R. (2021). A N-Acetyl-D-Galactosamine-Binding Lectin from Amaranthus Gangeticus Seeds Inhibits Biofilm Formation and Ehrlich Ascites Carcinoma Cell Growth in Vivo in Mice. Int. J. Biol. Macromol..

[32] Sharon N., Lis H. (1972). Lectins: Cell-Agglutinating and Sugar-Specific Proteins. Science.

[33] Rinderle S.J., Goldstein I.J., Remsen E.E. (1990). Physicochemical Properties of Amaranthin, the Lectin from *Amaranthus caudatus* Seeds. Biochemistry.

[34] Fang Y.Z., Yang S., Wu G. (2002). Free Radicals, Antioxidants, and Nutrition. Nutrition.

[35] Pham-Huy L.A., He H., Pham-Huy C. (2008). Free Radicals, Antioxidants in Disease and Health. Int. J. Biomed. Sci..

[36] Dimitrios B. (2006). Sources of Natural Phenolic Antioxidants. Trends Food Sci. Technol..

[37] Manach C., Scalbert A., Morand C., Rémésy C., Jiménez L. (2004). Polyphenols: Food Sources and Bioavailability. Am. J. Clin. Nutr..

[38] Samaranayaka A.G.P., Li-Chan E.C.Y. (2011). Food-Derived Peptidic Antioxidants: A Review of Their Production, Assessment, and Potential Applications. J. Funct. Foods.

[39] Repo-Carrasco-Valencia R. (2009). Dietary Fiber and Other Functional Components in Two Varieties of Crude and Extruded Kiwicha (*Amaranthus caudatus*). J. Cereal Sci..

[40] Chen H.M., Muramoto K., Yamauchi F., Fujimoto K., Nokihara K. (1998). Antioxidative Properties of Histidine-Containing Peptides Designed from Peptide Fragments Found in the Digests of a Soybean Protein. J. Agric. Food Chem..

[41] Elias R.J., Kellerby S.S., Decker E.A. (2008). Antioxidant Activity of Proteins and Peptides. Crit. Rev. Food Sci. Nutr..

[42] Nwachukwu I.D., Aluko R.E. (2019). Structural and Functional Properties of Food Protein-Derived Antioxidant Peptides. J. Food Biochem..

[43] Silva-Sánchez C., Barba De La Rosa A.P., León-Galván M.F., De Lumen B.O., De León-Rodríguez A., González De Mejía E. (2008). Bioactive Peptides in Amaranth (*Amaranthus hypochondriacus*) Seed. J. Agric. Food Chem..

[44] Ayala-Niño A., Rodríguez-Serrano G.M., González-Olivares L.G., Contreras-López E., Regal-López P., Cepeda-Saez A. (2019). Sequence Identification of Bioactive Peptides from Amaranth Seed Proteins (*Amaranthus hypochondriacus* spp.). Molecules.

[45] Vilcacundo R., Martínez-Villaluenga C., Miralles B., Hernández-Ledesma B. (2019). Release of Multifunctional Peptides from Kiwicha (*Amaranthus caudatus*) Protein under in Vitro Gastrointestinal Digestion. J. Sci. Food Agric..

[46] Xu D.P., Li Y., Meng X., Zhou T., Zhou Y., Zheng J., Zhang J.J., Li H. (2017). Bin Natural Antioxidants in Foods and Medicinal Plants: Extraction, Assessment and Resources. Int. J. Mol. Sci..

[47] Montoya-Rodríguez A., Milán-Carrillo J., Reyes-Moreno C., de Mejía E.G., González de Mejía E. (2015). Characterization of Peptides Found in Unprocessed and Extruded Amaranth (*Amaranthus hypochondriacus*) Pepsin/Pancreatin Hydrolysates. Int. J. Mol. Sci..

[48] Mir N., Riar C., Singh S. (2018). Nutritional Constituents of Pseudo Cereals and Their Potential Use in Food Systems: A Review.

[49] Kenmochi E., Kabir S.R., Ogawa T., Naude R., Tateno H., Hirabayashi J., Muramoto K. (2015). Isolation and Biochemical Characterization of Apios Tuber Lectin. Molecules.

[50] Banerjee S., Naresh M., Swamy M.J. (2024). Effect of Temperature and PH on the Structure and Stability of Tumor-Specific Lectin Jacalin and Insights into the Location of Its Tryptophan Residues: CD, DSC and Fluorescence Studies. Int. J. Biol. Macromol..

[51] Singh R.S., Thakur S.R., Kennedy J.F. (2020). Purification and Characterisation of a Xylose-Specific Mitogenic Lectin from Fusarium Sambucinum. Int. J. Biol. Macromol..

[52] e Lacerda R.R., do Nascimento E.S., de Lacerda J.T.J.G., Pinto L.d.S., Rizzi C., Bezerra M.M., Pinto I.R., Filho S.M.P., Pinto V.d.P.T., Filho G.C. (2017). Lectin from Seeds of a Brazilian Lima Bean Variety (*Phaseolus lunatus* L. Var. *cascavel*) Presents Antioxidant, Antitumour and Gastroprotective Activities. Int. J. Biol. Macromol..

[53] Vasconcelos I.M., Oliveira J.T.A. (2004). Antinutritional Properties of Plant Lectins. Toxicon.

[54] Solá R.J., Griebenow K. (2009). Effects of Glycosylation on the Stability of Protein Pharmaceuticals. J. Pharm. Sci..

[55] Kaur N., Dhuna V., Kamboj S.S., Agrewala J., Singh J. (2006). A Novel Antiproliferative and Antifungal Lectin from Amaranthus Viridis Linn Seeds. Protein Pept. Lett..

[56] Ahmad S., Khan R.H., Ahmad A. (1999). Physicochemical Characterization of Cajanus Cajan Lectin: Effect of PH and Metal Ions on Lectin Carbohydrate Interaction. Biochim. Biophys. Acta Gen. Subj..

[57] (2012). Para la Organización y Funcionamiento de los Laboratorios Clínicos.

[58] (2012). Para la Disposición de Sangre Humana y Sus Componentes con Fines Terapéuticos.

[59] Horwitz D.W., Latimer D.G. (2019). Official Methods of Analysis of AOAC International.

[60] Corke H., Cai Y.Y.Z., Wu H.X. (2016). Amaranth: Overview. Encyclopedia of Food Grains.

[61] Valadez-Vega C., Morales-González J., Sumaya-Martínez M., Delgado-Olivares L., Cruz-Castañeda A., Bautista M., Sánchez-Gutiérrez M., Zuñiga-Pérez C., Valadez-Vega C., Morales-González J.A. (2014). Cytotoxic and Antiproliferative Effect of Tepary Bean Lectins on C33-A, MCF-7, SKNSH, and SW480 Cell Lines. Molecules.

[62] Bradford M.M. (1976). A Rapid and Sensitive Method for the Quantitation of Microgram Quantities of Protein Utilizing the Principle of Protein-Dye Binding. Anal. Biochem..

[63] Basilio-Cortés U., González-Cruz L., Velazquez G., Teniente-Martínez G., Gómez-Aldapa C., Castro-Rosas J., Bernardino-Nicanor A. (2019). Effect of Dual Modification on the Spectroscopic, Calorimetric, Viscosimetric and Morphological Characteristics of Corn Starch. Polymers.

[64] Valadez-Vega C., Lugo-Magaña O., Betanzos-Cabrera G., Villagómez-Ibarra J.R. (2022). Partial Characterization of Lectins Purified from the Surco and Vara (Furrow and Rod) Varieties of Black Phaseolus Vulgaris. Molecules.

[65] Singleton V., Rossi J. (1965). Colorimetry of Total Phenolics with Phosphomolybdic-Phosphotungstic Acid Reagents. Am. J. Enol. Vitic..

[66] DuBois M., Gilles K.A., Hamilton J.K., Rebers P.A., Smith F. (1956). Colorimetric Method for Determination of Sugars and Related Substances. Anal. Chem..

[67] Federica T., Da Ros T., Passamonti S. (2012). Screening of Fullerene Toxicity by Hemolysis Assay. Methods Mol. Biol..

